# Lipid-based nutrient supplement at initiation of antiretroviral therapy does not substitute energy from habitual diet among HIV patients – a secondary analysis of data from a randomised controlled trial in Ethiopia

**DOI:** 10.29219/fnr.v66.5659

**Published:** 2022-02-11

**Authors:** Nanna Buhl Schwartz, Daniel Yilma, Tsinuel Girma, Markos Tesfaye, Christian Mølgaard, Kim Fleischer Michaelsen, Pernille Kæstel, Henrik Friis, Mette Frahm Olsen

**Affiliations:** 1 Department of Nutrition, Exercise and Sports, University of Copenhagen, Copenhagen, Denmark; 2 Department of Internal Medicine, Jimma University Specialized Hospital, Jimma, Ethiopia; 3 Department of Paediatric and Child Health, Jimma University Specialized Hospital, Jimma, Ethiopia; 4 Department of Psychiatry, St. Paul’s Hospital Millennium Medical College, Addis Ababa, Ethiopia; 5 Department of Infectious Diseases, Rigshospitalet, Denmark

**Keywords:** Africa, nutritional supplement, HIV, energy intake, food insecurity

## Abstract

**Introduction:**

Malnutrition is common among people with HIV in sub-Saharan Africa. Nutritional supplementation at initiation of antiretroviral treatment (ART) has shown beneficial effects, but it is not known if supplementation replaces or supplements the habitual energy intake in a context of food insecurity.

**Methods:**

As part of a randomised controlled trial among people with HIV initiating ART in Ethiopia, we assessed whether the provision of a lipid-based nutrient supplement (LNS) affected energy intake from the habitual diet. People with HIV aged ≥18 years with a body mass index (BMI) >17 were randomly allocated 2:1 to receive either early (month 1–3 after ART initiation) or delayed (month 4–6 after ART initiation) supplementation with LNS (≈4,600 kJ/day). Participants with BMI 16–17 were all allocated to early supplementation. The daily energy intake from the habitual diet (besides the energy contribution from LNS) was assessed using a 24-h food recall interview at baseline and at monthly follow-up visits. Linear mixed models were used to compare habitual energy intake in (1) early versus delayed supplementation groups and (2) supplemented versus unsupplemented time periods within groups.

**Results:**

Of 301 participants included, 67% of the participants were women, mean (±standard deviation [SD]) age was 32.9 (±8.9) years and 68% were living in moderately or severely food insecure households. Mean (±SD) reported habitual energy intake at baseline was 5,357 kJ/day (±2,246) for women and 7,977 kJ/day(±3,557) for men. Among all participants, there were no differences in mean habitual energy intake between supplemented and unsupplemented groups in neither the first 3 (*P* = 0.72) nor the following 3 months (*P* = 0.56). Furthermore, habitual energy intake did not differ within groups when comparing periods with or without supplementation (*P* = 0.15 and *P* = 0.20). The severity of food insecurity did not modify the effect of supplementation in habitual energy intake (*P* = 0.55). Findings were similar when participants with BMI 16–17 were excluded.

**Conclusion:**

Our findings indicate that the LNS provided after ART initiation supplement, rather than substitute, habitual energy intake among people with HIV, even among those who are food insecure. This supports the feasibility of introducing nutritional supplementation as part of HIV treatment.

## Popular scientific summary

- Three months of nutritional supplementation for people with HIV initiating antiretroviral treatment (ART) did not affect energy intake from their habitual diet in a food insecure setting in Ethiopia.
- Habitual energy intake was not substituted by the nutrition supplement, even among those living in severe food insecure households.
- Findings from this study support the feasibility of introducing nutritional supplementation as part of HIV care in settings with high food insecurity.

HIV remains a public health concern, especially in Eastern and Southern Africa, where 53% of the world’s 36.9 million people with HIV are living (1). Studies among people with HIV have shown that food insecurity and malnutrition increase the risk of opportunistic infections and poor treatment outcomes, including incomplete viral load suppression (2–4). In line with this, low body mass index (BMI) at initiation of antiretroviral treatment (ART) is an independent predictor of mortality among people with HIV in low-income settings (5–7). Weight loss in people with HIV is explained by a combination of increased energy requirements (8), inadequate energy uptake due to intestinal dysfunction and nausea after initiation of ART (9). In Ethiopia 12–42% of adults with HIV are underweight (BMI < 18.5 kg/m^2^) (10–13). According to WHO guidelines, nutritional assessment, counselling and support should be an integrated part of HIV care, especially in food insecure settings (14).

Several studies suggest that nutritional supplementation may support improved nutritional recovery and health outcomes among people with HIV in Sub-Saharan Africa (SSA) (15, 16). We have previously reported beneficial effects of 3 months supplementation with lipid-based nutrient supplement (LNS) on weight gain among people with HIV initiating ART. The weight gain mainly consisted of lean body mass and was accompanied by improved handgrip strength, immune recovery and quality of life (15, 17). However, to what extent a nutritional supplement replaces or in fact supplements the energy intake from the habitual diet has not been assessed among people with HIV in SSA. A few inconclusive studies have been reported from high-income countries (18–22), but to the best of our knowledge, no studies have assessed whether a nutritional supplement affects habitual energy intake among people with HIV in food insecure settings.

This information is highly relevant for assessing the feasibility of supplementation programmes in low-income settings. Based on data from our randomised controlled trial among people with HIV initiating ART in Ethiopia, we assessed whether the provision of LNS replaced or supplemented energy intake from the habitual diet.

## Methods

### Study population and design

**Fig. 1:**
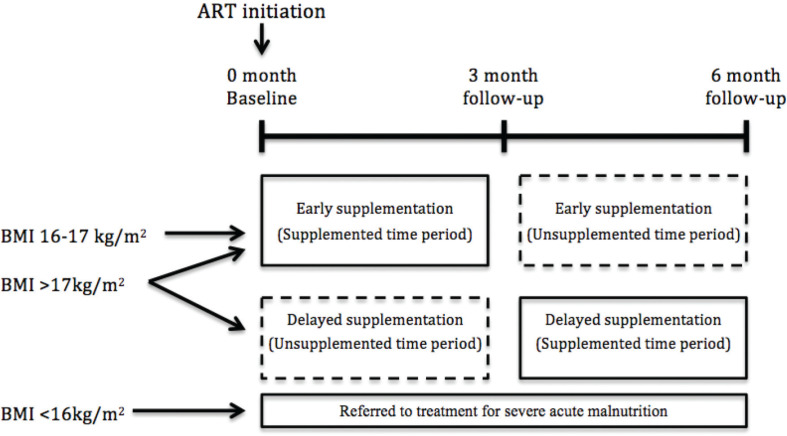
Study design and randomisation.

The ART food study was a randomised controlled trial conducted in Jimma, Ethiopia, between 2010 and 2013 (trial registration: ISRCTN32453477). As reported previously (15), participants with BMI > 17 were randomly allocated 2:1 to receive either early (month 1–3 after ART initiation) or delayed (month 4–6 after ART initiation) supplementation with a peanut-based LNS (Fig. 1). Participants with BMI 16–17 kg/m^2^ were all allocated to early supplementation, while participants with BMI <16 kg/m^2^ were excluded from the trial and referred to treatment for severe acute malnutrition following national guidelines (23). Participants were recruited at Jimma University Specialized Hospital (JUSH), Jimma Health Centre (JHC) and Agaro Health Centre in Oromia Region, Ethiopia. The inclusion criteria were age ≥18 years, BMI ≥ 16 kg/m^2^, living within a range of 50 km from recruitment facilities, HIV positive and eligible for initiation of ART. At the time the study was conducted, the Ethiopian national guideline for ART initiation was CD4 count ≤ 200 cells/ μL irrespective of clinical stage, CD4 count ≤ 350 cells/μL if WHO clinical stage III or WHO clinical stage IV regardless of CD4 count (24). The exclusion criteria were pregnancy, lactation or already taking micronutrient supplements or LNS. The sample size was calculated based on the trial’s primary outcome (lean body mass) (15). The availability of data from approximately 300 study participants for the present analysis allowed us to detect differences between early and delayed supplementation groups of 0.35 standard deviation [SD] with a 2:1 group ratio, 80% power and 5% significance level.

### Nutrition intervention

The nutritional supplement consisted of 200 g/day peanut-based LNS, developed by Nutriset (Malaunay, France). The supplement provided approximately half of the recommended daily energy intake (≈4,600 kJ) for the participants, and the energy distribution was 60% from fat, 16% from protein and 24% from carbohydrates. The micronutrient content of the supplement was 1–2 times the recommended nutrient intake from vitamins and minerals (25). Further details about the composition of the supplement have been published previously (15). The trial included a comparison of LNS with either soy or whey protein, but since we did not observe any difference in consumption, perception (26) or effects (15) related to the source of protein, the present analysis did not differentiate between supplements with soy and whey protein.

Participants were offered to try the LNS for 2 days before final enrolment. The supplement was distributed monthly and participants were asked to consume the supplement daily without sharing it with others. A pilot study had shown that the LNS supplements were acceptable and the daily amount perceived as realistic to consume over a period of 3 months (26). The ART food study included a qualitative assessment of perceptions and acceptability of LNS, which showed that the supplement was mainly consumed separately from other meals and was described by patients as part of their treatment rather than their habitual diet (26). Adherence to LNS was evaluated based on returned sachets and self-reported consumption and, as reported previously, adherence was high in both early and delayed supplementation groups (15).

### Data collection

**Fig. 2:**
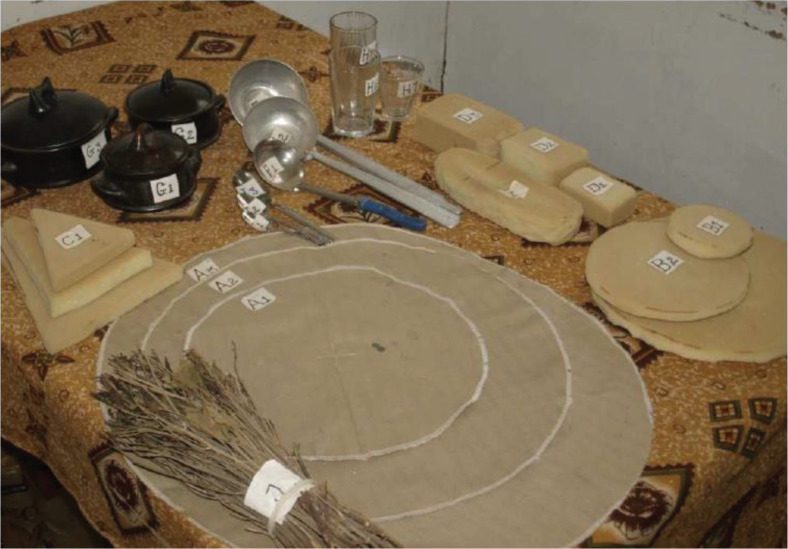
A locally produced recall kit with full-size models of traditional Ethiopian dishes.

Questionnaires were administered in the local languages Amharic and Afaan Oromo by trained study staff. Demographic characteristics such as age, sex, place of enrolment, education, religion and marital status were collected at baseline using a structured questionnaire. Height (SECA 214 Stadiometer Birmingham, UK) and weight (Tanita-BC 418 MA, Arlington Heights, USA) were measured barefoot while wearing minimal clothes. Information about WHO clinical stage of HIV and tuberculosis co-infection was obtained through the participants’ clinical records. A 24-h dietary recall interview was used to assess habitual energy intake from foods and beverages at baseline, and monthly follow-up visits up to 6 months. The energy intake from the habitual diet in the two time periods were calculated as the mean of the 24 h recall assessment at month 1, 2 and 3, and month 4, 5, and 6, respectively. A locally developed pre-coded chart and recall kit with full-size models of traditional Ethiopian dishes and household utensils were used to estimate portion size (Fig. 2). The participant’s energy intake from shared meals was calculated based on information about the number, age and sex of people sharing the meal. The energy content of the reported foods and beverages was assessed from standard recipes and local food composition tables (27, 28). The LNS was not considered part of the habitual energy intake. Food insecurity was assessed using the Household Food Insecurity Access Scale (HFIAS) (29) and categorised into food secure, mildly, moderately or severely food insecure, as described previously (30).

Potential under-reporting of daily energy intake was assessed by comparing the ratio between the estimated total energy expenditure (TEE) and the energy intake (EI) at baseline. As reported previously (30), TEE was calculated as the sum of physical activity energy expenditure (PAEE), resting energy expenditure (REE) and 10% diet-induced thermogenesis. Physical activity energy expenditure was measured with a combined uniaxial accelerometer and heart rate sensor (Actiheart, CamNtech, UK). Further details about PAEE assessment have been published previously (30). Resting energy expenditure was estimated by using the Oxford equation, including sex, age, weight and height (31) adding 10% due to the HIV infection (8).

### Data analysis

Stata/SE version 14.2 (StataCorp, College Station, USA) was used for statistical analysis. Continuous variables were assessed for normal distribution using histograms and normal probability quantile–quantile plots. The continuous variables with a normal distribution are presented as mean ± SD. Variables with a skewed distribution are presented as median with interquartile range (IQR). Categorical variables are presented as numbers and proportions.

Multiple linear regression analysis was used in a descriptive analysis of potential correlates of baseline energy intake, including demographic and anthropometric characteristics (sex, BMI, age, place of enrolment, education, religion, marital status, World Health Organization [WHO] clinical stage, tuberculosis co-infection and food insecurity). The analyses included two models: (1) unadjusted linear regression analysis and (2) adjusted for sex and age. The purpose of these analyses was to describe the participants’ habitual energy intake, to identify its correlates and to assess if our estimation of energy intake was sensitive enough to detect expected differences in a known-groups comparison.

A linear mixed model, including participant-specific random effects, was used to compare energy intake from specific food groups to investigate the potential differences in consumption patterns between early and delayed supplementation groups. The food groups were: (1) injera and bread, (2) other staples, (3) sauces (meat and vegetables), (4) porridge and soup, (5) dairy and egg, (6) fruit and juice, (7) drinks and (8) snacks (Supplementary Table 1).

In our primary analysis, linear mixed models, including participant-specific random effects, were used to compare habitual energy intake in (1) early versus delayed supplementation groups and (2) supplemented versus unsupplemented time periods within groups. Both unadjusted and adjusted models were fitted. Adjustment included potential confounders: age, sex, education, marital status, BMI groups and household food insecurity. It was evaluated if monthly visits could be summarised as supplemented and unsupplemented time periods (month 1–3 vs. month 4–6) by means of a test for interaction. In addition, a test for interaction between two subgroups for food insecurity (severely insecure vs. food secure or mildly insecure or moderately insecure) was performed to assess if the effect of supplementation on habitual energy intake was modified by the degree of household food security. Sensitivity analyses were conducted to assess whether the findings remained unchanged if excluding participants with (1) an average habitual energy intake outside 5th and 95th percentile, (2) BMI 16–17 kg/m^2^, or (3) adherence to LNS <90%. Finally, the degree of multicollinearity between variables was assessed using the variance inflation factor (VIF).

### Ethical considerations

The study was conducted in accordance with the Helsinki Declaration (32). Ethical approval was obtained from the Ethiopian National Health Research Ethical Review Committee (RDHE/30-90/2009) and Jimma University Ethical Review Board (15). Trial authorisation was obtained from the Food, Medicine and Health Care Administration and Control Authority of Ethiopia (02/6/05/50) and additional consultative approval from the Danish National Committee on Biomedical Research Ethics was obtained (15). Written informed consent was obtained from all participants. The study visits were organised on dates of ART care and transportation costs were reimbursed for all additional visits.

## Results

Of the 318 participants included in the ART food trial, data on habitual energy intake for at least two study visits were available for 301 participants. A flow chart of enrolment of participants and follow-up has been reported previously (15). Of the 301 participants included in the present analysis, 266 (88%) had a BMI > 17 kg/m^2^ of whom 179 (67%) were randomised to early supplementation and 87 (33%) to delayed supplementation. A total of 35 participants (12%) had a BMI between 16 and 17 kg/m^2^ and were allocated to early supplementation.

Information about sociodemographic characteristics and baseline energy intake is found in Table 1. Mean (±SD) age of included participants was 33.4 (±8.9) years and 31.6 (±8.7) years in the early and delayed supplementation groups, respectively. In both groups there were more women than men (65 and 71%), the majority of the participants lived in Jimma (JUSH and JHC) (78%), had some or completed level of either primary or secondary school (72 and 64%) and were widows or divorced (49 and 57%). The prevalence of severe household food insecurity was 41 and 36% in in early and delayed supplementation groups, respectively. In both groups, the majority of participants had a BMI ≥18.5 (56% and 71%), whereas only nine of all participants (3%) had a BMI ≥25. Baseline characteristics were similar when BMI 16–17 kg/m^2^ were excluded (*n* = 35, data not shown). No major differences with respect to baseline characteristics were observed between the early- and delayed supplementation.

**Table 1:** Baseline characteristics of 301 people with HIV allocated early or delayed nutritional supplementation after initiation of ART

Baseline characteristics	Early supplementation		Delayed supplementation	
	(*n* = 214)		(*n* = 87)	
**Age (years)**	33.4	[±8.9]	31.6	[±8.7]
**Age group**				
18–29 years	73	(34)	37	(43)
30–39 years	89	(42)	33	(38)
40+ years	52	(24)	17	(20)
**Sex (women)**	140	(65)	62	(71)
**Sex (Men)**	74	(35)	25	(29)
**BMI (kg/m** ^2^ **)**				
16–17 (*n* = 35)	35	(16)	0	(0)
17.1–18.4 (*n* = 85)	60	(28)	25	(29)
≥18.5 (*n* = 181)	119	(56)	62	(71)
**Place of enrolment**				
Jimma town (urban or semi-urban)	166	(78)	68	(78)
Agaro town (semi-urban or rural)	48	(22)	19	(22)
**Education**				
No formal schooling	59	(28)	31	(36)
Primary schooling^a^	112	(52)	40	(46)
Secondary school or higher^a^	43	(20)	16	(18)
**Religion**				
Islam	81	(38)	37	(43)
Christian orthodox	110	(51)	41	(47)
Protestant	23	(11)	9	(10)
**Marital status**				
Married	88	(41)	28	(32)
Widow or divorced	105	(49)	50	(57)
Single, never married or unknown	21	(10)	9	(10)
**Anthropometry**				
Weight (kg)	50.2	[±7.5]	50.6	[±7.7]
Height (cm)	160.9	[±8.4)	159.6	[±9.3]
BMI (kg/m^2^)	19.4	[17.5; 20.7]	19.8	[18.3; 21.0]
**WHO clinical stage**				
Stage I	71	(33)	25	(29)
Stage II	62	(29)	26	(30)
Stage III	65	(30)	28	(32)
Stage IV	16	(7)	8	(9)
**Tuberculosis co-infection**	24	(11)	7	(8)
**Household food security status** ^b^				
Secure	23	(11)	12	(14)
Mildly insecure	45	(21)	15	(17)
Moderate insecure	58	(27)	29	(33)
Severely insecure	88	(41)	31	(36)
**Baseline energy intake (kJ/day)**				
Women	5,359	[±2,295]	5,351	[±2,149]
Men	8,000	[±3,676]	7,910	[±3,247]

Note: Baseline characteristics are presented as *n* (%), mean [±SD] or median [IQR].

a Includes participants with some or complete level of education.

b Food insecurity was assessed using the Household Food Insecurity Access Scale.

### Habitual energy intake

The reported habitual energy intake at baseline was lower for women (5,357 kJ/day ± 2,246 SD) than men (7,977 kJ/day ± 3,557 SD) (*P <* 0.001) and participants living in the more rural setting of Agaro reported higher energy intake (7,405 kJ/day ± 3,469 SD) than participants living in Jimma (5,879 kJ/day ± 2,775 SD) (*P <* 0.001) (Table 2). Food secure participants had a higher energy intake (6,989 kJ/day ± 2,744 SD) than severely food insecure participants (5,377 kJ/day ± 2,602 SD) (*P* = 0.001) (Table 2). The observed difference between religious groups was explained by place of enrolment, as the proportion of Christian Orthodox was higher in Jimma (data not shown). No association was found between habitual energy intake and age groups, BMI groups, educational level, marital status, WHO clinical stage and tuberculosis co-infections after adjustment for sex and age.

**Table 2:** Baseline energy intake [kJ/day] for 301 people with HIV eligible for ART initiation by sociodemographic characteristics

Sociodemographic and clinical characteristics	*N*	Mean ± SD kJ/day	Model 1^a^		Model 2^b^	
			Coef. [95% CI]	*P*	Coef. [95% CI]	*P*
**Age group**						
18–29 years	110	5,791 ± 2,392	Ref.		Ref.	
30–39 years	122	6,503 ± 3,290	712 [−64; 1,488]	0.07	3 [−730; 737]	0.99
40+ years	69	6,398 ± 3,307	606 [−299; 1,512]	0.19	−557 [−1,439; 325]	0.22
**Sex**						
Male	99	7,977 ± 3,557	Ref.		Ref.	
Female	202	5,357 ± 2,246	−2,620 [−3,283; −1,957]	<0.001	−2,820 [−3,537; −2,104]	<0.001
**BMI (kg/m** ^2^ **)**						
16–17	35	5,699 ± 2,735	Ref.		Ref.	
17.1–18.4	85	6,170 ± 2,957	471 [−718; 1,660]	0.44	346 [−740; 1,433]	0.22
≥18.5	181	6,342 ± 3,081	643 [−450; 1,736]	0.25	597 [−400; 1,594]	0.24
**Place of enrolment**						
Jimma town (urban or semi-urban)	234	5,879 ± 2,775	Ref.		Ref.	
Agaro town (semi-urban or rural)	67	7,405 ± 3569	1,525 [723; 2,328]	<0.001	1,670 [944; 2,396]	<0.001
**Education**						
No formal schooling	90	5,676 ± 2,556	Ref.		Ref.	
Primary schooling^c^	152	6,314 ± 3,133	638 [−144; 1,420]	0.11	116 [−623; 855]	0.76
Secondary school or higher^c^	59	6,800 ± 3,210	1,124 [139; 2,109]	0.03	201 [−739; 1,142]	0.67
**Religion**						
Islam	118	6,692 ± 3,102	Ref.		Ref.	
Christian Orthodox	151	5,853 ± 2,762	−840 [−1,563; −117]	0.02	−665 [−1,327; −2.46]	0.049
Protestant	32	6,199 ± 3,560	−493 [−1,666; 679]	0.41	−318 [−1,394; 759]	0.56
**Marital status**						
Married	116	6,698 ± 3,022	Ref.		Ref.	
Widow or divorced	155	5,797 ± 2,879	−901 [−1,622; −180]	0.01	−204 [−895; 486]	0.56
Single, never married or unknown	30	6,544 ± 3,353	−154 [−1,357; 1,049]	0.80	−283 [−1,400; 835]	0.62
**WHO clinical stage**						
Stage I	96	6,421 ± 3,405	Ref.		Ref.	
Stage II	88	6,318 ± 2,850	−103 [−978; 772]	0.82	−395 [−1,195; 406]	0.33
Stage III	93	5,915 ± 2,683	−506 [−1,369; 357]	0.25	−642 [−1,431; 147]	0.11
Stage IV	24	6,226 ± 3,136	−195 [−1,548; 1,158]	0.78	−390 [−1,624; 845]	0.54
**Tuberculosis co-infection**						
No Tuberculosis infection	270	6,182 ± 2,989	Ref.		Ref.	
Tuberculosis infection	31	6,541 ± 3,180	359 [−763; 1,482]	0.53	228 [−800; 1,255]	0.66
**Household food insecurity status** ^d^						
Secure	35	6,989 ± 2,744	Ref.		Ref.	
Mildly insecure	60	7,035 ± 3,650	47 [−1,182; 1,275]	0.94	−336 [−1,462; 790]	0.56
Moderate insecure	87	6,498 ± 2,887	−491 [−1,647; 665]	0.40	−889 [−1,950; 171]	0.10
Severely insecure	119	5,377 ± 2,602	−1,612 [−2,723; −501]	0.01	−1,719 [−2,735; −702]	0.001

a Unadjusted linear regressions analysis of baseline energy intake.

b Multiple linear regression analysis of baseline energy intake adjusted for sex and age (years).

c Includes participants with some or complete level of education.

d Food insecurity was assessed using the Household Food Insecurity Access Scale.

The energy distribution by food group showed that injera or bread and sauces (meat or vegetables) were the dominating energy sources from the habitual diet (Table 3). No differences in consumption patterns were found between early- and delayed-supplementation groups (Supplementary Table 2).

**Table 3:** Habitual energy intake from food groups^a^ for 301 people with HIV allocated early or delayed nutritional supplementation after initiation of ART

Food groups	Early supplementation			Delayed supplementation		
	Energy kJ/day (Baseline) SD	Energy kJ/day (Month 1–3) SD	Energy kJ/day (Month 4–6) SD	Energy kJ/day (Baseline) SD	Energy kJ/day (Month 1–3) SD	Energy kJ/day (Month 4–6) SD
Injira and bread	3,486 ± 1,678	3,446 ± 1,624	3,616 ± 1,562	3,272 ± 1,811	3,476 ± 1,738	3,674 ± 1,650
Other staples	378 ± 847	313 ± 756	322 ± 778	498 ± 1,044	358 ± 854	325 ± 761
Sauces (meat and vegetables)	1,487 ± 1,315	1,346 ± 1,091	1,397 ± 1,022	1,349 ± 1,157	1,410 ± 1,037	1,446 ± 1,020
Porridge and soup	143 ± 513	185 ± 567	114 ± 427	147 ± 432	123 ± 499	196 ± 634
Dairy and egg	152 ± 411	115 ± 331	91 ± 289	81 ± 216	118 ± 426	113 ± 278
Fruit and juice	117 ± 302	151 ± 415	129 ± 336	159 ± 397	143 ± 349	95 ± 223
Drinks	162 ± 492	163 ± 492	192 ± 808	203 ± 594	165 ± 508	183 ± 905
Snacks	276 ± 395	336 ± 596	338 ± 485	322 ± 516	324 ± 470	275 ± 457

a Details of the eight food groups are in supplementary material (Supplementary Table 1).

Data from 209 participants (69%) were available for the comparison of the estimated TEE and the registered baseline EI. The EI/TEE ratio was 0.73 ± 0.34 SD (EI 27% lower than TEE) and 0.69 ± 0.31 SD (EI 31% lower than TEE) for men and women, respectively.

We found no interaction between early or late supplementation group and time period (*P* = 0.19), hence the differences between early versus late supplementation groups were assessed without taking time period into consideration. There were no differences in habitual energy intake (kJ/day) between those receiving and not receiving supplement in neither the first 3 (*P* = 0.72) nor the following 3 months (*P* = 0.56) after adjusting for age, sex, education, marital status, BMI group and household food insecurity (Table 4). Furthermore, no differences in habitual energy intake (kJ/day) between supplemented and unsupplemented time periods within groups were observed (*P* = 0.15 for early and *P* = 0.20 for delayed supplementation) (Table 4). A mean VIF value of 1.59 indicated a low and unproblematic degree of multicollinearity between variables. The association between food supplementation and habitual energy intake was not modified by the degree of food security (test of interaction *P* = 0.55). Sensitivity analyses showed that the exclusion of participants with BMI 16–17 kg/m^2^, those with an average energy intake outside 5th and 95th percentiles or those with supplement adherence <90% did not affect the results.

**Table 4:** Habitual energy intake^a^ among 301 adult people with HIV allocated early or delayed supplementation

Time period	Unadjusted ^b^			Adjusted^c^		
	Early supplementation (*n* = 214)	Delayed supplementation (*n* = 87)	Difference [95% CI]	Early supplementation (*n* = 214)	Delayed supplementation (*n* = 87)	Difference [95% CI]
	Mean SE	Mean SE		Mean SE	Mean SE	
Month 1–3: Energy intake kJ/day^d^	6,130 ± 163	6,120 ± 255	−9 [−603; 584] *P* = 0.98	6,102 ± 143	6,201 ± 227	99 [−434; 633] *P* = 0.72
Month 4–6: Energy intake kJ/day^e^	6,291 ± 166	6,350 ± 258	60 [−542; 661] *P* = 0.85	6,266 ± 146	6,429 ± 230	163 [−379; 705] *P* = 0.56
Difference [95% CI]	161 [−64; 386] *P* = 0.16	230 [−117; 577] *P* = 0.19		164 [−61; 389] *P* = 0.15	228 [−118; 574] *P* = 0.20	

a Habitual energy intake from the diet does not include the provided LNS supplementation.

b Linear mixed model, including participant-specific random effects, analysis of 24 h diet recalls for participants with early or delayed supplementation.

c Linear mixed model, including participant-specific random effects, analysis of 24 h diet recalls for participants with early or delayed supplementation. Estimates are adjusted for age, sex, education, marital status, BMI groups and household food insecurity.

d Average of 24 h diet recalls collected at end of month 1, 2, and 3.

e Average of 24 h diet recalls collected at end of month 4, 5, and 6.

## Discussion

The present study described habitual energy intake among people living with HIV at initiation of ART. The study found no differences in energy intake from habitual diet between those receiving and not receiving a nutritional supplement during the first 3 months, the following 3 months of ART, or between periods with and without supplementation within the same group. These results are supported by the observed weight gain during supplementation (15) and suggest that provision of LNS at initiation of ART does in fact supplement, rather than replace habitual energy intake. Furthermore, findings from our subgroup analyses support the feasibility of introducing nutritional supplementation to both food secure and insecure and moderately malnourished people with HIV without risking that the energy provided merely substitutes habitual intake.

### Strength and limitations

The main strength of this study is its design, which allowed us to compare changes in energy intake between groups receiving and not receiving supplementation and within groups before and after supplementation. The 24-h recall method for dietary intake assessment has been used widely and is considered a useful measure for assessment of dietary energy intake in groups or populations (33). However, the recall method is prone to recall bias and does not capture day-to-day variations. Under-reporting is likely to be present and the use of standard recipes from 1980 and 1998 to estimate energy content may have added to underestimation of energy intake, since fat content of common dishes has increased as a result of nutritional transition (34). Furthermore, despite the attempt to address meal sharing, this still imposed some imprecision in the assessment of individual habitual energy intake. Nevertheless, the reliability of our estimation of energy intake was strengthened by using the mean of three individual assessments. Although large variability of energy intake measurements may have reduced our ability to detect differences between groups, we note that data did allow us to detect expected differences in energy intake related to participants’ sex and food security.

We acknowledge that the data in this study were not collected recently, but we believe that our findings may still contribute to relevant information to support the feasibility of providing nutritional supplementation as part of HIV care. The availability of nutritional support in HIV care programmes across low-income countries has increased (35), but there is still a lack of data describing the influence of nutritional supplementation on the habitual energy intake in settings with high food insecurity.

### Other studies

To the best of our knowledge, only studies from high-income countries have previously assessed the effect of nutritional supplementation on habitual energy intake. Finding from these studies have been inconclusive: a 6 month nutrition trial among people with HIV in Switzerland, found an increase in total energy intake during the first 2 months of supplementation, followed by a reduction of habitual energy intake corresponding to the energy content of a supplement given (2,550 kJ/day) (18). A reduction of habitual energy intake was also observed in a trial in USA among people with HIV receiving nutritional supplementation of 2,350 kJ/day (19). Only a small increase in the total energy intake (habitual energy intake + supplementation) corresponding to 6–34% of the energy content of a fortified drink supplement (2,500 kJ/day) was observed in a trial in Germany suggesting a reduction of habitual energy intake (20). A small increase in total energy intake was observed during 3 months of supplementation with 3,100–3,800 kJ/day of people with HIV in Spain (22), while no significant increase in total energy intake was observed in a study among people with HIV in Switzerland receiving a supplement of 2,500 kJ/day, also suggesting a reduction of habitual energy intake (21).

In contrast to these studies, our trial was conducted in a low-income setting where two-thirds of the study population reported moderate or severe food insecurity. Energy deficiency was likely to be present, which may explain why our findings differ from previous observations in high-income settings. Possible differences in disease stage, treatment guidelines and type of supplementation may also explain the observed differences. A trial among malnourished HIV patients in Malawi found that LNS was less often shared than corn–soy blend, since it was seen as a special supplement for patients and did not require any preparation (36). This is supported by qualitative data from our study showing that participants perceived LNS as part of their HIV treatment and did not share with household members (26).

### Reliability of energy intake assessment

It is generally acknowledged that assessment of energy intake using dietary recalls tends to underestimate the actual energy intake and under-reporting has been reported in other studies with an EI substantially lower than TEE (37). In a review of doubly labelled water (DLW) validation studies, a combined analysis of 11 studies found an EI/TEE ratio of 0.87 ± 0.09 SD for men and 0.85 ± 0.10 SD for women and among the studies using 24-h dietary recall, the EI/TEE ratio was found to be 0.84 ± 0.08 SD (38). In the current analysis, the EI/TEE ratio at baseline was 0.73 and 0.69 for men and women, respectively, which may suggest a high degree of under-reporting. However, agreement between EI and TEE is not necessarily expected due to variation in the measurements (38). Furthermore, considering the advanced HIV status of the study participants at ART initiation and the prevalence of food insecurity, the observed difference between EI and TEE might indicate that the participants were undergoing weight loss at study inclusion.

As reported previously, the mean weight gain in the early supplementation group was approximately 2.7 kg of which a third consisted of lean body mass (15). Considering that it requires 33.5 kJ to synthesise one gram of adipose tissue and 7.5 kJ to synthesise one gram of lean tissue (39), it would require 67 MJ to gain the observed increase in lean and fat mass. Three months of the nutritional supplement provided participants with a total of 414 MJ. In addition to weight gain, the energy from supplementation contributed to covering the pre-existing deficiency in energy requirements, increased energy needs from tissue accretion and increased physical activity level after ART initiation (15). A more accurate estimate of energy balance is complex and includes additional factors such as malabsorption, increased energy requirement due to weight gain, diet-induced thermogenesis and infections.

In conclusion, this study found that LNS supplement adds to, rather than substitutes, habitual energy intake among people with HIV initiating ART even in a context of high food insecurity. These findings support the feasibility of introducing nutritional supplementation as part of HIV care programmes.

## Supplementary Material

Lipid-based nutrient supplement at initiation of antiretroviral therapy does not substitute energy from habitual diet among HIV patients – a secondary analysis of data from a randomised controlled trial in Ethiopia
