# Supplementary material for: Lipid-based nutrient supplement at initiation of antiretroviral therapy does not substitute energy from habitual diet among HIV patients – a secondary analysis of data from a randomised controlled trial in Ethiopia
Source: Food Nutr Res. 2022 Feb 11;66:10.29219/fnr.v66.5659. doi: 10.29219/fnr.v66.5659 (PMC8886435; doi:10.29219/fnr.v66.5659)
Supplement: Lipid-based nutrient supplement at initiation of antiretroviral therapy does not substitute energy from habitual diet among HIV patients – a secondary analysis of data from a randomised controlled trial in Ethiopia [file FNR-66-5659-s001.docx]

**Supplementary material**

| **Table S1:** Details of food groups | |
| --- | --- |
| Food group |  |
| 1. Injera and bread | Injera^1^, bread and false banana bread^2^ |
| 1. Other staples | Rice, spaghetti, kinche^3^, maize, potato and sweet potato |
| 1. Sauces (meat and vegetables) | Shiro^4^, tomato, missir^4^, siga^5^, kitfo^6^, tibs^6^ and firfir^7^ |
| 1. Porridge and soup | Genfo^8^ and shurba^9^ |
| 1. Dairy and egg | Milk, cheese and egg |
| 1. Fruit and juice | Banana, orange, avocado, mango, papaya, and other |
| 1. Drinks | Soft drinks, abish^10^, atmit^11^, tella keribo^12^, coffee/tea and beer |
| 1. Snacks | Qolo^13^, biscuit, butter, honey and sugar |
| ^1^Fermented flat bread, ^2^Bread made of fermented ensete stem, ^3^Boiled wheat or barley, ^4^ Sauces based on legumes and vegetables, ^5^Meat sauce, ^6^Dish with meat, ^7^ Source based on injera, ^8^Porridge, ^9^Soup, ^10^Fenugreek, ^11^Barley or wheat drink, ^12^Non-alcoholic barley drink, ^13^Roasted cereals or bread pieces | |

| **Table S2:** Differences in habitual energy intake by food groups for 301 people with HIV allocated early or delayed supplementation | | | | | | | |
| --- | --- | --- | --- | --- | --- | --- | --- |
|  | Unadjusted^1^ | |  |  | Adjusted^2^ | |  |
|  | Early  supplementation  (n =214) | Delayed  supplementation  (n = 87) |  |  | Early  supplementation  (n =214) | Delayed  supplementation  (n = 87) |  |
|  | Coef. [95% CI] | Coef. [95% CI] | *p* |  | Coef. [95% CI] | Coef. [95% CI] | *p* |
| **Injera and bread**  Energy kJ/day (Month 1-3)  Energy kJ/day (Month 4-6)  **Other staples**  Energy kJ/day (Month 1-3)  Energy kJ/day (Month 4-6)  **Sauces (meat and vegetables)**  Energy kJ/day (Month 1-3)  Energy kJ/day (Month 4-6)  **Porridge and soup.**  Energy kJ/day (Month 1-3)  Energy kJ/day (Month 4-6)  **Dairy and egg**  Energy kJ/day (Month 1-3)  Energy kJ/day (Month 4-6)  **Fruit and juice**  Energy kJ/day (Month 1-3)  Energy kJ/day (Month 4-6)  **Drinks**  Energy kJ/day (Month 1-3)  Energy kJ/day (Month 4-6)  **Snacks**  Energy kJ/day (Month 1-3)  Energy kJ/day (Month 4-6) | -19 [-341; 303]  Reference  -43 [-165; 78]  Reference  -49 [-251; 152]  Reference  62 [-18; 141]  Reference  -1 [-58; 57]  Reference  7 [-51; 66]  Reference  -1 [-111; 109]  Reference  11 [-78; 100]  Reference | Reference  46 [-282; 373]  Reference  3 [-122; 127]  Reference  31 [-174; 237]  Reference  83 [2; 165]  Reference  16 [-42; 75]  Reference  -34 [-94; 27]  Reference  -12 [-125; 101]  Reference  -63 [-154; 27] | 0.91  0.78  0.49  0.97  0.63  0.76  0.13  0.05  0.98  0.58  0.81  0.27  0.98  0.84  0.81  0.17 |  | -69 [-368; 229]  Reference  -40 [-164; 84]  Reference  -96 [-276; 83]  Reference  67 [-14; 148]  Reference  7 [-50; 63]  Reference  1 [-58; 61]  Reference  -9 [-120; 102]  Reference  21 [-68; 109]  Reference | Reference  95 [-209; 398]  Reference  -4 [-131; 123]  Reference  77 [-106; 261]  Reference  78 [-5; 161]  Reference  7 [-50; 65]  Reference  -28 [-89; 33]  Reference  -3 [-117; 111]  Reference  -73 [-163; 18] | 0.65  0.54  0.53  0.96  0.29  0.41  0.11  0.07  0.82  0.80  0.97  0.37  0.88  0.96  0.65  0.11 |
| ^1^Linear mixed model, including participant-specific random effects, analysis of 24 hour diet recalls for participants with early or delayed supplementation.  ^2^Linear mixed model, including participant-specific random effects, analysis of 24 hour diet recalls for participants with early or delayed supplementation. Estimates are adjusted for age, sex, education and marital status, BMI groups and household food insecurity | | | | | | | |
